# The impact of pulse timing on cortical and subthalamic nucleus deep brain stimulation evoked potentials

**DOI:** 10.3389/fnhum.2022.1009223

**Published:** 2022-09-20

**Authors:** Brett A. Campbell, Leonardo Favi Bocca, David Escobar Sanabria, Julio Almeida, Richard Rammo, Sean J. Nagel, Andre G. Machado, Kenneth B. Baker

**Affiliations:** ^1^Department of Biomedical Engineering, Case Western Reserve University, Cleveland, OH, United States; ^2^Department of Neurosciences, Lerner Research Institute, Cleveland Clinic, Cleveland, OH, United States; ^3^Center for Neurological Restoration, Neurological Institute, Cleveland Clinic, Cleveland, OH, United States; ^4^Department of Biomedical Engineering, Lerner Research Institute, Cleveland Clinic, Cleveland, OH, United States; ^5^Department of Neurosurgery, Neurological Institute, Cleveland Clinic, Cleveland, OH, United States

**Keywords:** deep brain stimulation, evoked potentials, pulse timing, Parkinson' disease, beta

## Abstract

The impact of pulse timing is an important factor in our understanding of how to effectively modulate the basal ganglia thalamocortical (BGTC) circuit. Single pulse low-frequency DBS-evoked potentials generated through electrical stimulation of the subthalamic nucleus (STN) provide insight into circuit activation, but how the long-latency components change as a function of pulse timing is not well-understood. We investigated how timing between stimulation pulses delivered in the STN region influence the neural activity in the STN and cortex. DBS leads implanted in the STN of five patients with Parkinson's disease were temporarily externalized, allowing for the delivery of paired pulses with inter-pulse intervals (IPIs) ranging from 0.2 to 10 ms. Neural activation was measured through local field potential (LFP) recordings from the DBS lead and scalp EEG. DBS-evoked potentials were computed using contacts positioned in dorsolateral STN as determined through co-registered post-operative imaging. We quantified the degree to which distinct IPIs influenced the amplitude of evoked responses across frequencies and time using the wavelet transform and power spectral density curves. The beta frequency content of the DBS evoked responses in the STN and scalp EEG increased as a function of pulse-interval timing. Pulse intervals <1.0 ms apart were associated with minimal to no change in the evoked response. IPIs from 1.5 to 3.0 ms yielded a significant increase in the evoked response, while those >4 ms produced modest, but non-significant growth. Beta frequency activity in the scalp EEG and STN LFP response was maximal when IPIs were between 1.5 and 4.0 ms. These results demonstrate that long-latency components of DBS-evoked responses are pre-dominantly in the beta frequency range and that pulse interval timing impacts the level of BGTC circuit activation.

## Introduction

Deep brain stimulation (DBS) for Parkinson's disease (PD) involves chronic high-frequency stimulation within nodes of the basal-ganglia-thalamocortical (BGTC) circuit, including the subthalamic nucleus (STN) (Limousin et al., 1998; Deuschl et al., 2006). Our understanding of the mechanism of action of DBS remains poorly understood and attempts at improving DBS or translating it to other disease modalities could benefit from a better understanding of how to effectively modulate circuit dynamics. Manipulating pulse timing and capitalizing on the effects of temporal summation provides a means of enhancing circuit engagement without increasing stimulation amplitude and current spread, which can be associated with undesirable side-effects (Matsumoto et al., 2008). Furthermore, a better understanding of temporal dynamics may improve our ability to develop new technology that targets BGTC circuit modulation to deliver therapeutic benefit and treat disease.

Low-frequency DBS-evoked potentials (DBS-EPs) provide insight into circuit activation that may further be linked to both therapeutic benefit and side-effects (Schmidt et al., 2020). Recent studies on DBS-EPs have focused heavily on the short-latency peaks occurring within the period of traditional DBS (<7 ms) and demonstrated their potential significance in the therapeutic efficacy of STN-DBS (Li et al., 2007, 2012; Dejean et al., 2009; Walker et al., 2012). However, the long-latency components may also be of value and have even been tied to motor side effects associated with traditional DBS for PD (Romeo et al., 2019; Irwin et al., 2020). No study has yet demonstrated how the timing of multiple pulses may modulate the frequency content or long-latency (>7 ms) components of DBS-EPs. This information may help to improve our fundamental understanding of how to modulate the BGTC circuit and inform next-generation DBS paradigms that rely on effective delivery of pulse sequences to alter circuit dynamics.

Closed-loop stimulation often utilizes firing patterns that rely on bursts of pulses in order to up- or down-regulate relevant physiological biomarkers or pathophysiology (Tass, 2003; Rosin et al., 2011; Adamchic et al., 2014; Johnson et al., 2016). One such biomarker in PD is the increased beta band (13–35 Hz) power observed in spontaneous resting state recordings made across nodal points in the BGTC circuit (Brown, 2003). Recent studies have demonstrated that stimulation-evoked activity measured at the site of stimulation, such as globus pallidus internus (GPi), shows oscillatory activity consistent with the peaks in the power spectral density observed during resting state recordings (Escobar Sanabria et al., 2022). Those same studies have demonstrated that phase-locking this evoked oscillatory activity to spontaneous beta activity can allow for direct control of beta oscillations that may be useful for understanding their role in PD (Escobar Sanabria et al., 2020, 2022; Fleming et al., 2020). Therefore, an understanding of how the temporal aspects of stimulation can influence the evoked beta oscillations can improve our ability to utilize this closed-loop stimulation approach to control neural activity in real-time.

We investigated the effects of pulse timing on circuit activation as measured through LFP recordings made from the STN DBS lead as well as from scalp EEG in the form of DBS-EPs. The response profile was characterized by measuring the impact of electrical pulses on the frequency content of the evoked responses. This characterization helped us to identify how stimulation-evoked beta band oscillations changed as a function of pulse timing to provide a fundamental understanding of how pulse timing impacts this aspect of modulation of the BGTC circuit.

## Methods

### Data acquisition and analysis

#### Participants

All research was approved by the Cleveland Clinic Institutional Review Board and participants provided signed informed consent (NCT04563143). Data were derived from individuals who underwent staged, bilateral STN DBS implants, with only the DBS lead implanted as part of the second, staged surgical procedure externalized for up to 10 days prior to IPG placement to allow for recoding and stimulation. Data were collected between days three and eight of the externalization period, with the participant awake and semi-reclined in a cushioned chair or hospital bed. Acquisition was performed while the patients who were on anti-parkinsonian medication were actively on their medication with the exception of participant one. Pre-operative clinical data was obtained from medical records, with OFF-medication MDS-UPDRS part III scoring performed and recorded by a movement disorders neurologist as standard of care practice at the Cleveland Clinic.

#### Surgical procedure

All participants underwent awake lead implantation following conventional, frame-based stereotactic technique. Pre-operative MRI and CT head imaging were obtained for trajectory planning. Patients received sedation and anesthesia monitoring and local anesthesia prior to head fixation of a stereotactic Leksell frame model G (Elekta, Sweden). Frame registration was performed using an O-arm scan (Medtronic, Minneapolis, MN, USA) set to stereotaxy protocol. For stereotactic coordinates, the StealthStation S8 System (Medtronic, Minneapolis, MN, USA) was used for pre-operative and intraoperative imaging co-registration with the trajectory targeting the ventral border of STN and skull entry made anterior to the coronal suture. Intraoperative microelectrode recordings and low dose O-arm scans were used to assess the trajectory. Macrostimulation was used for stimulation side effects. Final lead position was tested for symptom improvement and side-effect threshold. For this study, the implanted lead was connected to a conventional extension and its distal connector externalized.

#### Anatomical localization of leads

**Figure 1:**
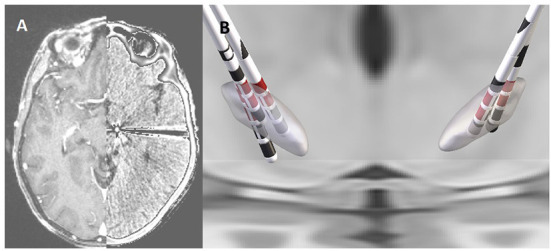
Co-registered pre-operative MRI and post-operative CT scan from an individual participant **(A)**. Reconstruction of the DBS leads in the STN for each of the five participants with the stimulation contacts shown in red **(B)**.

Each patient underwent pre-operative MRI (volumetric T1-weighted with contrast) and post-implant CT, which were used for anatomical verification of lead position. All DICOM file images were converted to NIfTI file format using the dcm2niix protocol. The implanted leads were visually-localized using CT, which was co-registered to pre-operative T1 MRI using a two-stage linear registration (rigid followed by affine) as implemented in Advanced Normalization Tools (Figure 1A) (Avants et al., 2008). Pre-operative acquisitions were spatially normalized into MNI ICBM 2009b non-linear asymmetric space (Fonov et al., 2011) using a three-step non-linear affine registration (Avants et al., 2008; Schönecker et al., 2009). Localization of each contact of the DBS lead was done post-operatively using MATLAB toolbox Lead DBS v2.5.3, following methodology described elsewhere (Horn et al., 2019). After image processing, the Cartesian coordinates were measured at the geometric center of each contact referenced to AC-PC midpoint and expressed in millimeters with one decimal place of accuracy (x, y, and z, respectively lateral, anterior and below the midcommissural point). The DISTAL brain atlas was used for target segmentation (Ewert et al., 2018). The dorsal-lateral region of the STN for each participant was visually identified by a clinician in the co-registered imaging. The contacts that were positioned within or directly facing dorsal-lateral STN were then identified.

#### Contact selection

All participants underwent mono- and bi- polar review of the externalized lead on the first post-operative day. During review, a constant rate of 130 Hz and pulse width of 90 μs were used, with the exception of participant five where a pulse width of 60 μS was used. For each lead contact, the amplitude of stimulation was increased in constant increments, with side-effect(s) threshold, localization, subjective intensity (low, medium, high), and duration (transient or continuous) documented. The maximum amplitude of stimulation with transient side-effects was considered as the maximum tolerable threshold of stimulation. The efficacy of stimulation on rigidity and bradykinesia was assessed for multiple contact configurations at increasing pulse amplitudes by a clinician consistent with conventional clinical practice (Volkmann et al., 2006). The active contact used (cathode selection) for subsequent electrophysiological evaluation was chosen based on both imaging criteria (the contacts in the closest position relative to dorsolateral region of STN as identified through the process of lead localization described above; see Figure 1) and also presenting the largest stimulation therapeutic window available.

#### Stimulation settings and pulse delivery

**Figure 2:**
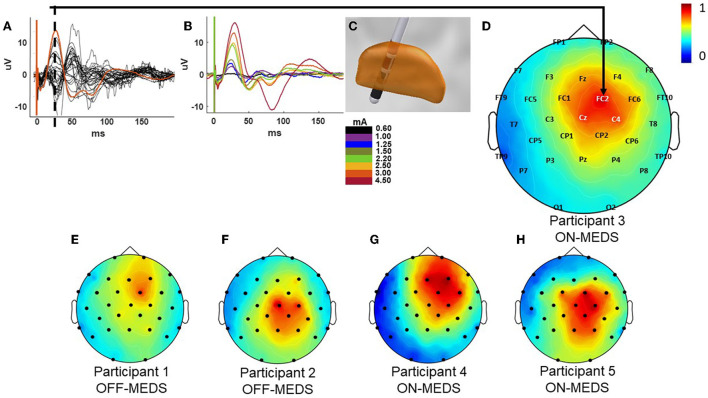
Example cortical evoked potential response and the current source density (CSD) plots for all participants. Butterfly plot of the EEG recordings during stimulation at 3mA with the FC2 electrode shown in orange from participant 3 while on medication **(A)**. Cortical evoked response across the FC2 electrode site at increasing stimulation amplitudes from the same participant **(B)**. Co-registration of the DBS lead in the STN showing the stimulation contacts in red generating the responses for participant 3 shown in parts A–D **(C)**. CSD plot at the 25 ms time point post the onset of stimulation indicated by the dotted line in A for participant 3 **(D)**. The CSD plots for participants 1 & 2 [off-medication; **(E,F)**] and 4 & 5 [on-medication; **(G,H)**]. All CSD plots are normalized and reflect the left-right flip to align to a common (right) side.

**Figure 3:**
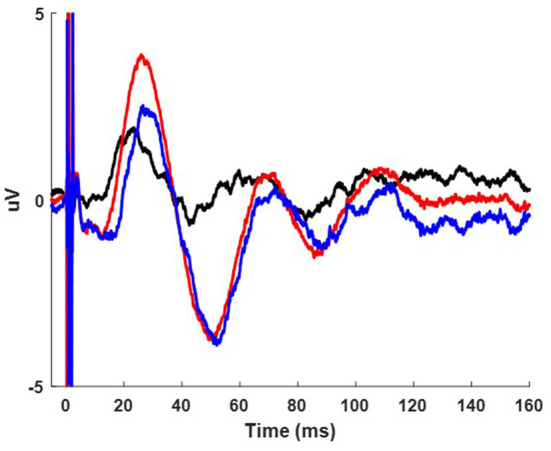
Example cortical evoked potential response during the paired pulse condition from participant 3 while on medication from the FC2 electrode. The 1.5 ms IPI (red) condition is shown overlaid on the single pulse condition (black) and resulting cortical waveform from subtracting the single pulse condition from the paired pulse condition (blue).

A symmetric biphasic square-wave pulse was used and delivered in a bipolar configuration using the Subject Interface Module from Tucker Davis Technologies (Alachua, FL, USA). All patients underwent stimulation with pulse width of 90 μs and with amplitude set to a clinically effective point as determined through the bipolar review across the selected contact pair. Side effects were ruled out at high frequency stimulation (i.e., 130 Hz) at the chosen amplitude before undergoing the low-frequency stimulation at that same amplitude. Lead and extension integrity were confirmed by impedance check, with all values below 3 kΩs. An amplitude sweep was performed with a single pulse ranging from 0.60 to 4.50 mA across the selected stimulation contacts to confirm that the chosen amplitude generated an evoked response whose amplitude fell within the linear portion of the response growth curve and showed no evidence of saturation that might mitigate any pulse-timing effect (Figure 2). Paired-pulse stimulation was then performed to evaluate the effect of pulse timing on the EP by sending two pulses—a conditioning pulse followed by a test pulse of equal stimulation intensity that varied only in timing relative to the first (see Figure 3). The test pulse occurred at specific inter-pulse intervals (IPI: 0.2, 0.5, 0.7, 0.8, 1.0, 1.5, 2.0, 2.5, 3.0, 4.0, 5.0, 7.7, 8.3, and 10 ms) and the sequence for delivery of the 14 IPIs was randomized. A total of 750 pulses were delivered for the single-pulse and each paired-pulse IPI (a conditioning pulse followed by a test pulse) at 5.1 Hz frequency to create the average EP. The single-pulse condition was performed at the beginning and end of the randomized IPI sequence as well as periodically throughout to use as a single pulse baseline. The composite average was visualized with a five-second delay during acquisition using MATLAB v 2021a (Mathworks, Natick, MA, USA) and Synapse from Tucker Davis Technologies (Alachua, FL, USA) to monitor for artifacts and allow for trial rejection and additional sample acquisition for each IPI as needed.

#### Signal collection and processing

A total of 30 silver/silver chloride electrodes were adhered to the scalp according to the traditional 10–20 configuration, with additional electrodes placed at FC1, FC2, FC5, FC6, FT9, CP1, CP2, CP5, CP6, TP9, and TP10. Data were collected using equipment from Tucker Davis Technologies and sampled at 24,414.06 Hz with an anti-aliasing filter set at 45% of the sampling rate and processed using custom scripts on the Cleveland Clinic high-performance computer. We re-referenced the EEG data with respect to the average across EEG channels and data were left-right flipped to align to a common (right) side. The evoked potential was computed by averaging data segments aligned with the first stimulation artifact. To remove the DC offset from the EP, a 0.5 ms baseline period prior to the stimulation pulse was subtracted. A 10-point moving average filter was employed to remove high-frequency noise. The single-pulse was aligned with the timing of the conditioning pulse and subtracted from each IPI condition to derive the differential effect of the second pulse relative the test pulse (de Goede et al., 2020). Quantification was limited to the C4, FC2, Cz, and CP2 electrode recording sites given their proximity to the motor region of the cortex as shown in the current source density plot in Figure 2D, and was only performed for electrodes ipsilateral to STN stimulation. This choice was data-driven upon preliminary examination of the CSD maps across participants that showed sensor-level activity localized to those regions consistently. To increase the frequency resolution the EPs were zero padded. We blanked the electrical artifact for 2.5 ms in the EEG data to reduce artifact contamination. The frequency content with respect to time was assessed using Morse continuous wavelet transform (CWT) after applying a second order band pass filter between 8 and 100 Hz to remove low and high frequency noise (Wachowiak et al., 2018). The single pulse cortical EP and corresponding wavelet for each participant from their FC2 electrode can be seen in the Supplemental Figure 1. The average wavelet amplitude was computed within a window of 6 Hz bandwidth and 50 ms around the maximum wavelet amplitude observed in the EP for quantification and statistical comparison of the effect of each IPI on the resulting EP (see statistics section). We performed the work in this study using the Fieldtrip toolbox (Oostenveld et al., 2011) and visualized the current source density plots using the Brainstorm toolbox (Tadel et al., 2011) as well as custom scripts in MATLAB.

The Welch's power spectral density (PSD) (Welch, 1967) was computed to characterize the frequency distribution of the EP. The frequency power was computed as the sum of PSD values in alpha, low beta, high beta, low gamma, and high gamma (8–12, 13–20, 21–35, 36–70, and 71–100 Hz, respectively) to evaluate changes within each frequency band due to different IPIs. Seven and a half minutes of resting state data were collected under the same conditions as the EP data and were used to normalize the PSD of the EP data and to allow for grouping across participants. Normalization was done by creating a ratio of the PSD from the EP over the resting state PSD values. The normalization also allowed for an appreciation of the EP power compared to the power of spontaneous activity in the same recording site across different frequency bands.

Recordings were also made from the remaining contacts of the DBS lead not used for stimulation using the same reference/ground configuration noted above. Post-acquisition the STN recording channels were differentially re-referenced to create an LFP and a single channel pair was selected for each participant for analysis to maintain a consistent sample size given the variability in the amount of available recording channels with each participant. Pseudo-annular rings were created across rows of directional contacts for some participants, which meant that only two contacts (dorsal and ventral most contacts) remained available for recoding and thus served as the differential reference for those participants. When multiple channels were available, the configuration with the largest response post differential re-referencing was selected. The single pulse condition and corresponding wavelet for the STN-EP for each participant is shown in the Supplemental Figure 2. Stimulation artifact was removed using a template subtraction approach (Escobar Sanabria et al., 2022). The sampling rate (≈24 KHz), wide-band of the antialiasing filter (45% of the ≈24 KHz sampling rate), and the amplifier input range (+/-) 500 mV allowed for capturing the artifact shape without ringing confounds. The averaged STN-EP was initially computed using the same method as for cortical channels with reversed anodal-cathodal phase-order configurations collected to facilitate determination of the starting point for the physiological response. Artifacts were characterized by a short-latency, high-frequency component followed by a low-frequency drift. The process of creating the artifact template is as follows:

1) Obtain and visually inspect the STN-EP to verify a neural response.
2) Determine the sample point (*n*_0_) when short-latency artifacts end.
3) Choose samples throughout the STN-EP to interpolate the template model to capture the neural responses and low-frequency drift.
4) Interpolate from sample (*n*_0_) to the end of the STN-EP using a shape-preserving piecewise cubic interpolation (PCHIP method in MATLAB, Mathworks, Natick, MA).
5) To include the short-latency artifacts in the template for removal alongside the low-frequency drift the first (*n*_0_) samples of the STN-EP were included as a part of the template.

The artifact template was built for each of the IPI configurations and subtracted from their corresponding averaged STN-EP to obtain the final waveform for analysis. An example of the template removal can be seen in the Supplemental Figure 3. Data from the DBS lead was otherwise assessed using the methods described above for cortical EEG data once the template was subtracted.

### Statistics

To determine whether the presence of the conditioning pulse influenced the response compared to the single pulse condition, permutation statistics were performed on an individual subject. This method utilized up to 750 pulses prior to averaging and subtraction to observe the effect on the EP wavelet amplitude on a trial-by-trial basis. Permutation statistics were done between the single pulse condition and the 0.2, 2.5, and 10 ms IPI to capture differing levels of potential augmentation. The difference in the amplitude of the EPs for each IPI compared to the single pulse condition was characterized using spectrograms calculated with the wavelet transform as described above. We assessed whether the differences in EP values in the time-frequency domain were the result of chance using a permutation test with replacement (Good, 1994), performed as follows:

1) Create a set of surrogate EP segments combine all segments from each of the two conditions evaluated.
2) Randomly partition the surrogate EP segments into two sets assigned to their respective IPI conditions.
3) Create average EP surrogates for each condition, calculate the wavelet spectrogram for each, and compute the absolute value of the difference between the wavelet spectrograms at each time and frequency.
4) Repeat steps 1–3 to create a set of 5,000 spectrogram differences (surrogate distribution).
5) Using the permutation distribution of the surrogate spectrogram differences, we computed the *p-*value of the original difference value at each frequency and time.
6) Apply a false discovery rate (FDR) correction in the time and frequency domains to the *p-*values to account for the multiple tests performed in the time and frequency domains.

**Figure 4:**
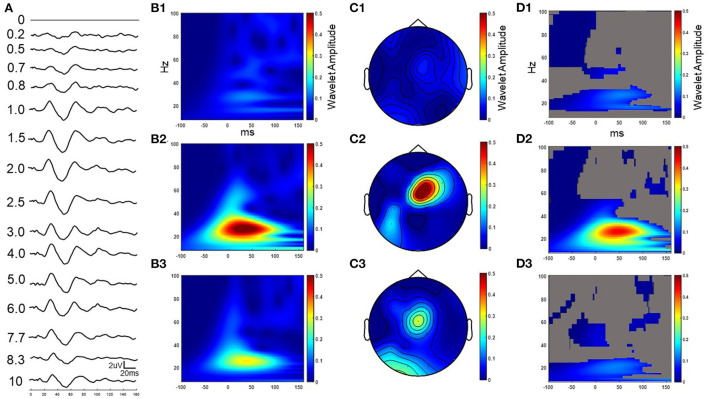
Example cortical evoked response (FC2) to increasing IPIs from participant 3 while on medication. The result in the time domain post subtraction of the single pulse from the paired pulse at increasing IPIs **(A)**. The wavelet response of the time domain subtracted 0.2, 2.5, and 10 ms IPI conditions from column A (**B1–B3** respectively). Wavelet spatial localization of the 0.2, 2.5, and 10ms IPI conditions from column A (**C1–C3** respectively). Permutation statistics comparing wavelet amplitude differences in the frequency domain between the single pulse and non-subtracted paired pulses at the 0.2, 2.5, and 10 ms IPIs with non-significant time-frequency zones shown in gray (**D1–D3** respectively). Z scales are wavelet amplitudes and time zero is aligned to the stimulation artifact with plots showing frequencies between 8-100 Hz.

The resulting spectrogram reflects the average difference in wavelet amplitude between the single pulse and paired pulse condition. An example is presented in Figure 4D. Non-significant values from the permutation statistics were represented with gray areas on the spectrograms. Significant regions were values with *p* < 0.05. The results from these permutation statistics provided the rationale for extracting the average wavelet amplitude from within a defined region of maximal activation for the purposes of group analysis.

A window with 6 Hz bandwidth over 50 ms and centered at the maximum wavelet amplitude in the EP spectrogram was then extracted and averaged to provide a single scalar value for each subject at each IPI for further statistical comparison at a group level. The bandwidth and time for this extraction was based on the findings from the permutation statistics from individual participant analysis. In order to normalize and group subject data, an amplitude ratio was created of the extracted average spectrogram value at each IPI over the average value for the single pulse condition. This was done for channels FC2, C4, CP2, and Cz as well as the STN-LFP. A ratio value >1 reflects an augmented response, while a value <1 indicates a reduction. A value of one indicates that the conditioning pulse did not alter the test-pulse EP. The data were matched and compared against a value of one using Friedman's test and Dunn's multiple comparison test. No Gaussian distributions were assumed and *p-*values are presented. The tests were two-tailed with an alpha of 0.05 and performed using GraphPad Prism v9 (GraphPad Software, San Diego, CA, USA).

The PSD of the EP for each IPI, including the single pulse condition, was normalized to the resting state as described above for the FC2, C4, CP2, CZ, and STN LFP. The frequency content for the normalized PSD ratio at each band (alpha, low beta, high beta, low gamma, and high gamma) across each IPI was compared to the normalized PSD ratio for the single pulse condition. The data were matched and evaluated using Friedman's test and Dunn's multiple comparison test. No Gaussian distributions were assumed and *p-*values are presented. The tests were two-tailed with an alpha of 0.05 and also performed using GraphPad Prism v9.

## Results

### Participant demographics

The five participants had a mean age of 69.2 ± 5.9 years, a disease duration of 7.4 ± 2.9 years at the time of surgery, and two were female. The mean pre-operative, OFF-medication total MDS-UPDRS was 43.4 points and the mean levodopa equivalent replacement dose was 777.5 mg (Table 1). The mean post-operative improvement in MDS-UPDRS score after overnight withholding of medication and off stimulation was 18%. The final DBS lead location was grouped in the posterior part of STN, with all having at least two rows of contacts implanted within the target region (Figure 1B). All contacts selected for stimulation were deemed to be inside the target based upon co-registered imaging.

**Table 1:** Participant details.

**Subject**	**Age (years)**	**Disease Duration (years)**	**Sex**	**Implant Side**	**Off Medication Pre-operative UPDRS Score**	**Off Medication Post-operative UPDRS Score**	**Relative Change (percentage)**	**Pre-operative levodopa Equivalent Daily Dose**	**Position of Cathode** **(AcPc coord., mm)**			**Stimulation Amplitude (mA)**	**Lead Type**	**Lead Manufacturer**
									**X**	**Y**	**Z**			
1	74.3	9	Male	left	55	30	−25(−45.5)	1360	−12	−3.5	2.5	3	DB-2202	Boston Scientific
2	76	8	Male	Right	52	52	0	0	13.8	−0.7	1	3	6172	St. Jude
3	62.5	4	Female	Right	33	47	14(+42.4)	1095	10.3	−1.3	2.2	0.9	B33005	Medtronic
4	68.4	11	Female	left	25	10	−15(−60)	422.5	−10.9	−1.8	2	2.5	B33005	Medtronic
5	64.7	5	Male	Right	52	38	−14(−26.9)	1010	12.6	−2	2.6	1.5	B33005	Medtronic

### Single-pulse activation

Low-frequency single-pulse STN stimulation elicited a cortical EP that was prominent across the hemisphere ipsilateral to STN stimulation and with long-latency components that contained the largest consistent positive deflection at 25 ms post the onset of stimulation (Figure 2). The response was maximal across the motor and premotor regions of the cortex as revealed by the butterfly plot and CSD plots of the cortical EP (Figure 2A) and the corresponding current source density plots (Figures 2D–H). Components of the EP grew as a function of increasing pulse-amplitude up to the maximum pulse amplitude of 4.5 mA (Figure 2B). The EP lasted up to 150–200 ms post the onset of stimulation at the largest stimulation amplitudes tested (2.20–4.50 mA).

### Paired-pulses enhance beta activity in the cortex and STN

A series of paired pulses at 14 different IPIs were delivered in the STN (Figure 3). The arrival of the conditioning pulse produced changes in the EP in the motor region of the cortex as shown through the response across the FC2 electrode location in Figure 4. As IPIs increased from 0 (i.e., the gray, single-pulse condition) to 10 ms the resulting waveform showed prominent oscillations beginning as early as 20 ms after the onset of stimulation. Conditioning pulses arriving 1.5–2.0 ms prior showed the largest response of all the tested IPIs (Figure 4A). Conditioning pulses arriving < 0.8 ms had little to no effect on the test EP. The wavelet of the cortical evoked response from 100 ms pre- to 150 ms post-stimulation showed the largest oscillatory response occurring within the beta band (Figures 4B1–B3). The smallest IPI of 0.2 ms showed minimal augmenting effect, which is visible both in the waveform in Figure 4A as well as in the wavelet response shown in Figure 4B1. As shown in Figure 4B2, the wavelet amplitude was maximal around the 2.5 ms IPI paring and occurred within the first 100 ms from stimulation onset. As the IPI increased to 10 ms the effect of the conditioning pulse on the resulting EP was reduced as shown by the decrease in the wavelet amplitude of the response (Figure 4B3). The wavelet response profile localized to the motor regions of the cortex ipsilateral to the site of stimulation as shown in Figures 4C1–C3 for the 0.2, 2.5, and 10 ms IPIs, respectively. When comparing the 0.2 ms IPI to the single pulse condition using permutation statistics, significant differences in the frequency content are centered in the beta and gamma regions (α < 0.05; 5,000 permutations; Figure 4D1). This finding was also present in the 2.5 and 10 ms IPI conditions (α < 0.05; 5,000 permutations; Figures 4D2,D3). The largest difference of those three, however, can be observed when the IPI is at 2.5 ms with a wavelet amplitude reaching its maximum peak at 50 ms after the onset of stimulation (Figure 4D2).

**Figure 5:**
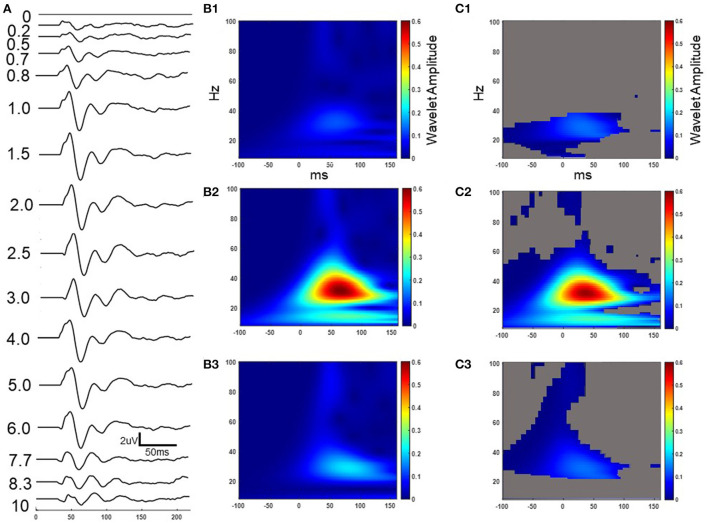
Example STN evoked response to increasing IPIs from participant 3 while on medication. The result in the time domain post subtraction of the single pulse from the paired pulse at increasing IPIs **(A)**. The wavelet response of the time domain subtracted 0.2, 2.5, and 10 ms IPI conditions from column A (**B1–B3** respectively). Permutation statistics comparing wavelet amplitude differences in the frequency domain between the single pulse and non-subtracted paired pulses at the 0.2, 2.5, and 10 ms IPIs with non-significant time-frequency zones shown in gray (**C1–C3** respectively). Z scales are wavelet amplitudes and time zero is aligned to the stimulation artifact with plots showing frequencies between 8-100 Hz.

The IPI also influenced the magnitude of the response recorded within the STN (Figure 5A). The single pulse condition showed oscillatory activity that persisted for the first 100 ms after the onset of stimulation. The augmenting effect of the conditioning pulse was minimal for IPIs < 0.8 ms apart (Figure 5A). However, the intensity of the EP grew and became most pronounced for those 0.7 ms and larger. After 6.0 ms, the compounding effect diminished compared to IPIs with shorter periods. The wavelet response at 0.2, 2.5, and 10 ms IPIs showed activation in the beta frequency range (Figures 5B1–B3, respectively). The beta component centered at 50 ms after the onset of stimulation across each IPI and changed only in amplitude as a function of changes in the IPI period, with the 2.5 ms condition producing the largest amplitude response. When comparing the 0.2 ms IPI to the single pulse condition using permutation statistics, the results demonstrate that a significant difference can be observed only in the beta frequency band (α < 0.05; 5,000 permutations; Figure 5C1). However, as the IPI increased to 2.5 ms the significant frequency regions included portions of the alpha and gamma bands, and expanded beyond the initial beta peak at 50 ms (α < 0.05; 5,000 permutations). The 10 ms IPI permutation statistics showed a difference only in the beta and gamma regions (α < 0.05; 5,000 permutations). No significant findings were observed in the alpha band. We also observed a decrease in the temporal range reaching significance around the 50 ms post the onset of stimulation similar to the 0.2 ms condition (α < 0.05; 5000 permutations).

**Figure 6:**
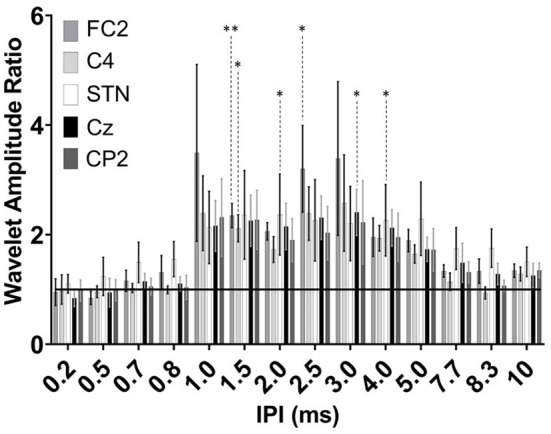
Group results of wavelet amplitude ratio for the FC2 (dark gray), C4 (light gray), STN-LFP (white), Cz (black), and CP2 (dark gray) at the 0.2–10 ms IPIs. Ratio reflects each subtracted IPI over the single pulse condition. Enhancement results in a ratio >1 and attenuation <1 with the horizontal black line at a ratio value of one. Significant differences are denoted by the asterisks with *P* < 0.05 and *P* < 0.001 (single and double asterisks, respectively) with error bars reflecting the standard error of the mean.

Extraction of the average maximum wavelet amplitude for each IPI was used to identify the pulse timing that had the greatest enhancing effect (Figure 6). Overall, the significant augmenting effects in the EEG were observed when pulses were delivered between 1.5 and 3.0 ms apart. No difference was observed in the wavelet amplitude ratio when pulses were spaced as close as 0.2–0.8 ms. At 1.5 ms, the enhancement was statistically significant compared to the single pulse condition for both the FC2 electrode location as well as C4 (*p* = 0.007 and *p* = 0.02, respectively). The response was also significantly different for the FC2 electrode at 2.5 ms (*p* = 0.020). For the Cz electrode a significant difference was observed when the IPI was at 3.0 ms (*p* = 0.03). No differences were identified in the CP2 electrode. The STN channel showed a significant difference between the single pulse and both the 2.0 and 4.0 ms IPI (*p* = 0.02; *p* = 0.03, respectively). Both the cortical and STN responses showed similar, but non-significant excitation when the IPI exceeded 4.0 ms as compared to delivery of a single pulse.

**Figure 7:**
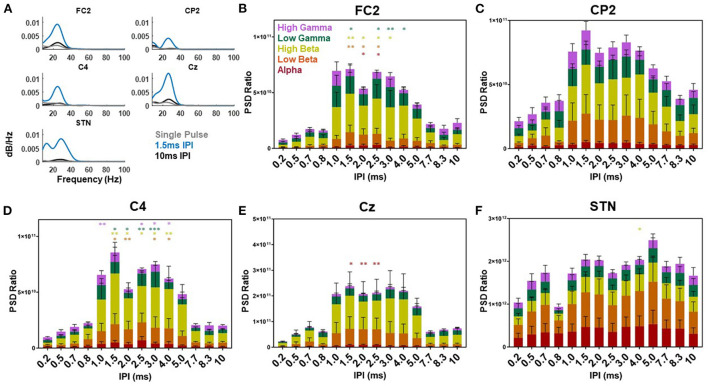
Single participant and group power spectral density results. Example power spectral density of the EP waveform for the single (gray), 1.5 (blue), and 10 ms (black) IPI across the FC2, CP2, C4, Cz, and STN-LFP channels from participant three while on medication **(A)**. The power spectral density ratio of the cortical EP from the FC2, CP2, C4, Cz, and STN-LFP channel with each IPI over the resting state power differentiated based on the average alpha (red), low beta (orange), high beta (yellow), low gamma (green), and high gamma (purple) frequencies across all participants (**B–F**, respectively). Significant differences are denoted by the single and double asterisks (*P* < 0.05 and *P* < 0.001, respectively) with error bars reflecting standard error of the mean.

Evaluation of the PSD of the cortical and STN EPs compared to the resting state PSD demonstrated the largest increase in power within the beta band as a function of the IPI (Figure 7). Across the STN-LFP, C4, CP2, Cz, and FC2 electrode locations the majority of the power in the single pulse EP (gray lines) was located in the beta band (shown for an individual participant in Figure 7A). The delivery of the second pulse increased the overall power of the EP across all three locations with minimal shift in the distribution (blue and black lines). When normalized to the resting state, the power in the FC2 EP increased as a function of increasing the IPI with the greatest increases observed when IPIs were between 1.0 and 5.0 ms (Figure 7B). In the alpha frequency band, a significant difference was observed between the 2.0 and 2.5 ms IPI and the single pulse condition (*p* = 0.03 and *p* = 0.02, respectively). The low beta frequency band showed a significant difference between the 1.5, 2.0, and 2.5 ms IPI and the single pulse condition (*p* = 0.004, *p* = 0.02, and *p* = 0.01, respectively). The high beta frequency band showed a significant difference between the 1.5, 2.0, 2.5, and 3.0 ms IPI and the single pulse condition (*p* = 0.009, *p* = 0.04, *p* = 0.02, and *p* = 0.02, respectively). Finally, the low gamma band showed significant differences between the 1.5, 2.5, 3.0, and 4.0 ms condition (*p* = 0.03, *p* = 0.02, *p* = 0.004, *p* = 0.03, respectively). While CP2 showed a trend toward increased power across frequency bands, this change did not reach statistical significance (Figure 7C).

Across the C4 location the PSD ratio was also the greatest when IPI were between 1.0 and 5.0 ms. The low beta band (13–20 Hz) showed a significant difference when the IPI was at 1.5, 2.0, 3.0, and 4.0 ms compared to the single pulse condition (*p* = 0.02, *p* = 0.005, *p* = 0.01, and *p* = 0.02, respectively). In high beta (21–35 Hz) the 1.5, 2.0, 2.5, 3.0, and 4.0 ms IPI showed a significant difference compared to the single pulse condition (*p* = 0.005, *p* = 0.02, *p* = 0.01, *p* = 0.01, and *p* = 0.009, respectively). Low gamma (36–70 Hz) was significantly different when IPIs were at 1.5, 2.0, 2.5, and 3.0 ms compared to the single pulse condition (*p* = 0.01, *p* = 0.03, *p* = 0.003, *p* = 0.003, and *p* < 0.001, respectively). High gamma was also modulated by the IPI at 1.0, 2.5, 3.0, and 4.0 ms compared to the single pulse condition (*p* = 0.007, *p* = 0.03, *p* = 0.03, and *p* = 0.03, respectively). For Cz the PSD showed a significant difference in the alpha band at the 1.5, 2.0, and 2.5 ms IPI (*p* = 0.03, *p* = 0.009, and *p* = 0.005, respectively). The PSD of the STN EP showed increases at all IPI compared to the single pulse condition. However, only the 4.0 ms IPI showed a significant difference compared to the single pulse condition (*p* = 0.03). This difference was in the high beta band (21–35 Hz). The remaining IPIs were not significantly different.

## Discussion

We investigated the effects of pulse timing on modulation of the BGTC circuit at the level of the STN using DBS-EPs and demonstrated that inter-pulse intervals consistent with high-frequency (i.e., >250 Hz) stimulation can have a significant impact on physiological activity recorded both at the level of the cortex and in the STN region. Specifically, the magnitude of the response observed at both levels was sensitive to IPI between the “condition” and “test” pulse pair, with those within the 1.0 to 3.0 ms range yielding enhanced activation (extending to 4.0 ms for recordings made at the STN). Values outside of that range, including IPIs in the range of traditional DBS, yielded minimal or no change in the test pulse response relative to the single-pulse condition.

Evoked potentials recorded both at the level of the cortex and from DBS lead recordings have been established as a physiological response to STN-DBS and used as a potential tool to inform therapeutic mechanisms and clinical programming. In preclinical work, both short- and long-latency EP components have been observed at the cortical level and shown to be sensitive to therapeutic *versus* non-therapeutic stimulation, disease severity, and the presence of dopaminergic medication (Li et al., 2007, 2012; Dejean et al., 2009; Campbell et al., 2021). In human studies, DBS-EPs have been recorded using both EEG and electrocorticography (ECoG) (Ashby et al., 1999, 2001; Baker et al., 2002; MacKinnon et al., 2005; Eusebio et al., 2009; Kuriakose et al., 2010; Walker et al., 2012; Miocinovic et al., 2018). The shorter latency components measured over the cortex, in both preclinical (0.5–2 ms) and clinical (2–5 ms) contexts, have been proposed as reflecting antidromic activation of the hyperdirect pathway between the motor cortex and STN. This hypothesis has been supported by modeling studies, which further suggest that activation of this pathway alone may be sufficient to generate the corresponding long-latency components (Kumaravelu et al., (2018); Bower and McIntyre, 2020; Gunalan and McIntyre, 2020; Bingham and McIntyre, 2022). In evaluating the potential use of DBS-EPs as a clinical tool, others have shown that STN-DBS EPs measured across the cortex can predict motor side effects from stimulation and may be a useful biomarker for optimizing DBS (Gmel et al., 2015; Romeo et al., 2019; Peeters et al., 2021; Sand et al., 2021). Finally, evoked potentials recorded from the STN have also demonstrated potential therapeutic use through modulation of the amplitude and frequency of the short-latency components in response to therapeutic stimulation parameters (Sinclair et al., 2019). Our study further supports their utility for optimizing the temporal elements of stimulation by showing changes in the DBS-EP long-latency components as a function of pulse timing measured both at the cortex and in the STN.

Paired pulse studies are a well-established method for evaluating inhibitory and facilitatory mechanisms (Kujirai et al., 1993; Kobayashi and Pascual-Leone, 2003; Prescott et al., 2013). A prior paired pulse study evaluated the short-latency components (<10 ms) in the STN, pallidum, and thalamus across Parkinson's disease and essential tremor patients and argued that DBS paired pulses can facilitate short-term plasticity and enhance communication between nodes in the BGTC circuit (Awad et al., 2021). Their findings suggest that the short-latency evoked resonant activity (occurring before 10 ms) in the STN was maximal when IPIs were >1 ms apart and peaked around 2 ms. We demonstrated that changes in long-latency components in the STN-EP were significant when pulses were spaced 2 ms apart, suggesting that IPIs in that range may best facilitate neural pathways responsible for both the short and long latency components in the STN-EP. Other studies have found that motor evoked potentials (MEPs) generated by TMS that are preceded by a DBS pulse 2.0–4.0 ms prior can maximally facilitate the MEP compared to other IPIs in Parkinson's disease patients (Hanajima et al., 2004; Ni et al., 2019). All of these findings combined with the results shown in this study suggest that IPIs centered on 2.0 ms may provide an ideal window for temporal summation of neural activity in the cortex and STN, and facilitate engagement of the BGTC circuit without the need to increase pulse amplitude to levels that risk spreading current beyond the targeted deep brain region.

A common approach in analyzing paired pulse studies is to subtract the single pulse condition from the paired pulse condition in the time domain. This practice, however, can produce latency shifts in the peak components due to the phase lag between the two oscillatory signals. The results that we present in Figures 4A–C, 5A,B show the time domain subtraction results, which could be impacted by latency differences between the two signals. One way to account for such effects is to transform the two signals to the frequency domain prior to any subtraction to avoid phase lag effects. The result of this approach is shown in Figures 4D1–D3, 5C1–C3, where we compare the wavelet response between conditions as this would not be impacted by latency changes (i.e., phase lag). In this case the transformation to the frequency domain is done first, followed by subtraction, which allows for the strength of the oscillations to be compared independent of their phase. Therefore, any differences between columns B and D in Figure 4 (and columns B and C of Figure 5) represent the influence of latency shifts in the time domain subtracted signal. This suggests that further research is needed to understand whether the long-latency components are pre-dominantly mediated by the similar mechanisms that underlie the short-latency components given that larger IPIs begin to attenuate and shift the response back to the single pulse condition.

A better understanding of the temporal elements of pulse delivery may help to refine adaptive stimulation paradigms that aim to increase the therapeutic window of STN-DBS. Bursting paradigms can be useful in avoiding undesirable current spread outside of the therapeutic region by minimizing the amplitude of stimulation while capitalizing on the effects of temporal summation. However, previous studies comparing regular and non-regular patterns as well as ON/OFF cycling of high frequency stimulation for PD showed differing levels of clinical efficacy despite each paradigm achieving the same average high rate of stimulation (Montgomery, 2005; Brocker et al., 2013). A similar observation has been made in relation to thalamic DBS for essential tremor, with Birdno et al. reporting that pulse spacing and not just the average rate of stimulation was a significant factor in the therapeutic efficacy (Birdno et al., 2007). The results from this study show that in order to maximize the response yielded in the motor cortex from intermittent STN stimulation pulse pairings should fall between 1.0 and 3.0 ms. Therefore, if the goal of a non-traditional or adaptive paradigm (i.e., bursting, evoked interference DBS, or phase targeting) is based on maximizing the downstream effects of pulses delivered relative to specific events or physiological activity as opposed to chronic isochronal stimulation, then delivering two pulses (per the significant time intervals reported here) would be most effective at facilitating that effort. Future studies should explore whether that does in fact translate to an improved benefit for patients receiving those non-traditional approaches given that such a claim is beyond the scope of the data reported in this study.

One closed-loop approach that may benefit from the results of this study is evoked interference DBS (eiDBS) that relies upon delivering individual electrical pulses with precise amplitude and timing relative to spontaneous physiological activity at the site of stimulation (Escobar Sanabria et al., 2020, 2022). In this approach stimulation is phase locked to beta band oscillations in the BGTC circuit. The evoked beta response from stimulation causes constructive or destructive interference with the spontaneous beta activity thereby allowing for amplification or suppression of beta activity in the BGTC circuit. This approach delivers stimulation at a lower frequency than traditional DBS. Therefore, the use of multiple pulses in quick succession with the proper IPI may facilitate more effective control of neural activity when a single pulse is insufficient in generating enough of an evoked response to generate the necessary interference to effectively suppress or amplify spontaneous beta power.

## Limitations

Three out of five participants were on their anti-Parkinson's disease medication during this study. One participant did not take anti-Parkinson's medication and another was evaluated off-medication after it was withheld overnight. Proper lead implantation also produces transitory improvement in disease severity and may hinder the generalization of reported results to long-term, chronically implanted patients (Lange et al., 2021). The differences observed in the average maximum wavelet amplitude in EEG channels over the cortex may be reflective of the location of axons with varying diameters relative to the DBS lead channels selected for stimulation. As shown in Figure 6, the 1.0 ms IPI showed a large increase in excitation, but failed to reach statistical significance. The variability in the FC2, CP2, Cz, and C4 electrodes at that IPI may have been due to the proximity of large diameter fibers to stimulation contacts in a few patients as opposed to others and their recruitment or lack thereof contributed to the non-significant finding at that IPI. Differences in lead placement may also help to explain some of the variability in both the cortical as well as STN response given that the contact chosen for stimulation in each participant was selected based on the findings from image co-registration to determine contacts best positioned in dorsal-lateral STN and motor thresholds.

## Data availability statement

The raw data supporting the conclusions of this article will be made available by the authors, without undue reservation.

## Ethics statement

The studies involving human participants were reviewed and approved by Cleveland Clinic Institutional Review Board. The patients/participants provided their written informed consent to participate in this study.

## Author contributions

BC, AM, and KB: study design. RR, SN, and AM: performed surgery. BC, LF, and JA: performed experiments. BC, LF, DE, and JA: analyzed data. BC, DE, AM, and KB: interpreted results. BC, LF, and JA: prepared figures. BC: drafted manuscript. All authors contributed to the article and approved the submitted version.

## Funding

Financial support was provided by the Farmer Family Foundation.

## Conflict of interest

AM and KB have intellectual property and distribution rights in Enspire and Cardionomics. AM has distribution rights in ATI and is a consultant to Abbott. SN consults for Abbott and is a speaker for Medtronic. The remaining authors declare that the research was conducted in the absence of any commercial or financial relationships that could be construed as a potential conflict of interest.

## Publisher's note

All claims expressed in this article are solely those of the authors and do not necessarily represent those of their affiliated organizations, or those of the publisher, the editors and the reviewers. Any product that may be evaluated in this article, or claim that may be made by its manufacturer, is not guaranteed or endorsed by the publisher.
