# Supplementary figures and images for: The impact of pulse timing on cortical and subthalamic nucleus deep brain stimulation evoked potentials
Source: Front Hum Neurosci. 2022 Sep 20;16:1009223. doi: 10.3389/fnhum.2022.1009223 (PMC9532054; doi:10.3389/fnhum.2022.1009223)

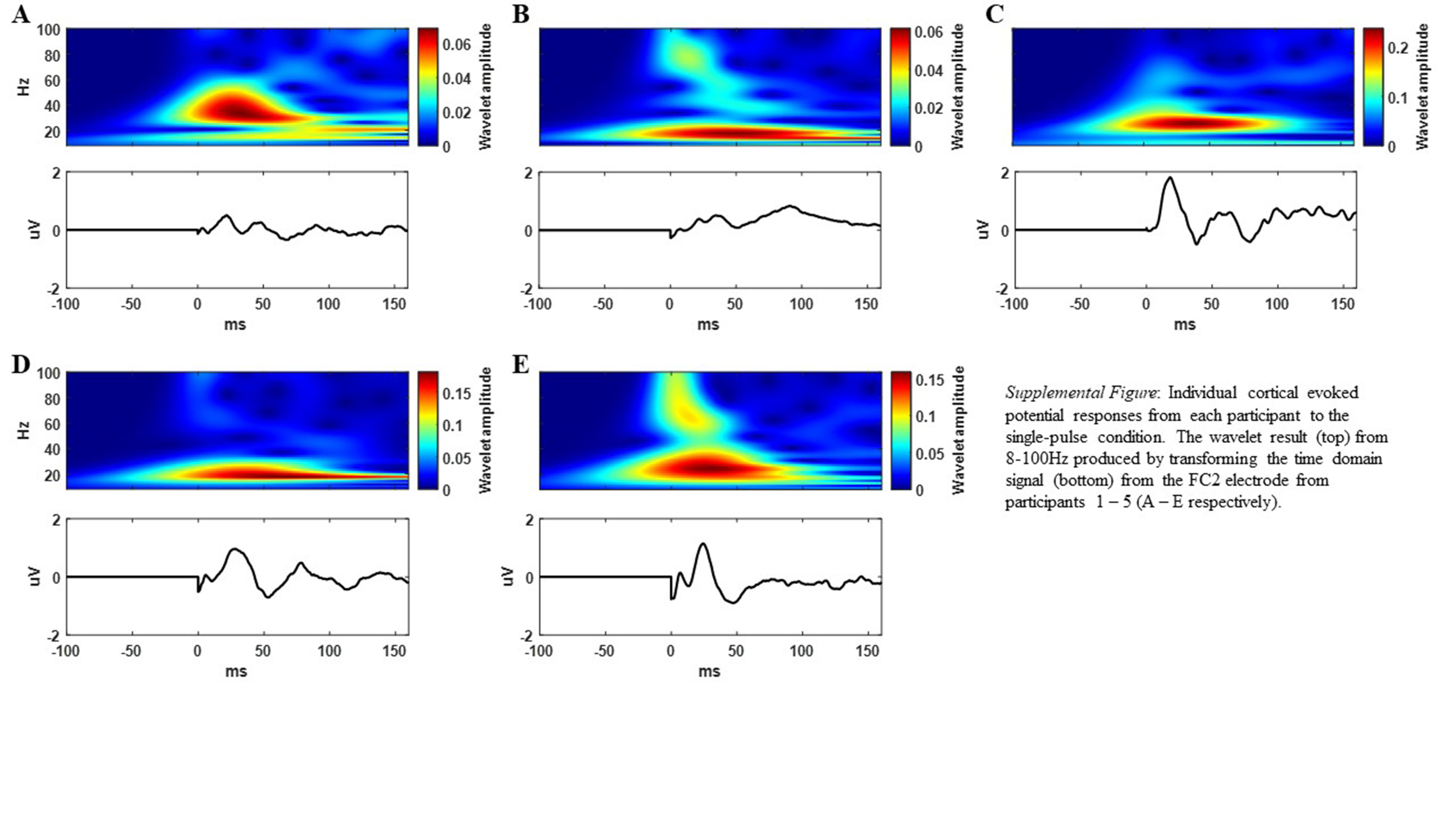

Supplement: Supplementary file 1 [file Image_1.JPEG]

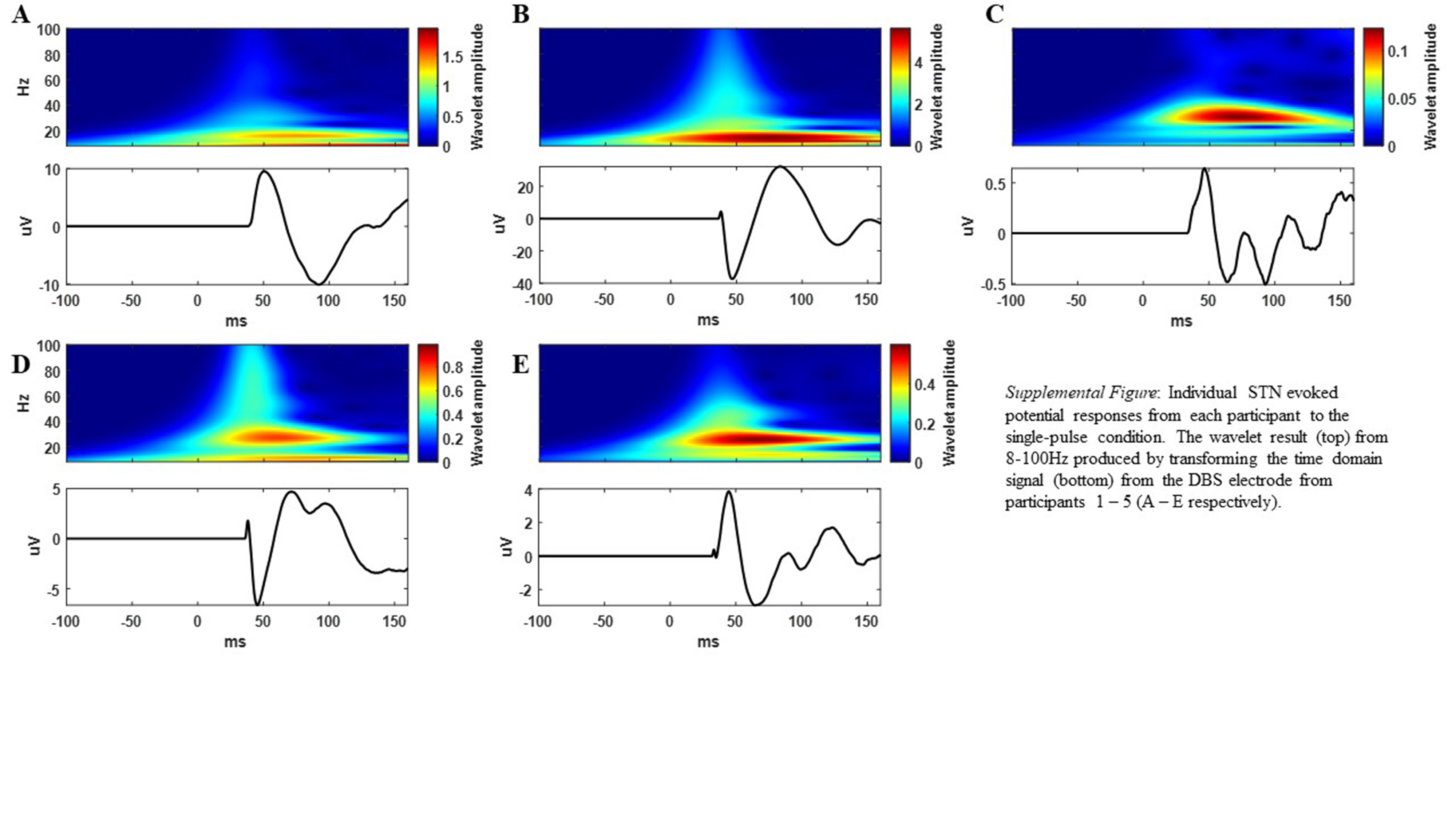

Supplement: Supplementary file 2 [file Image_2.JPEG]

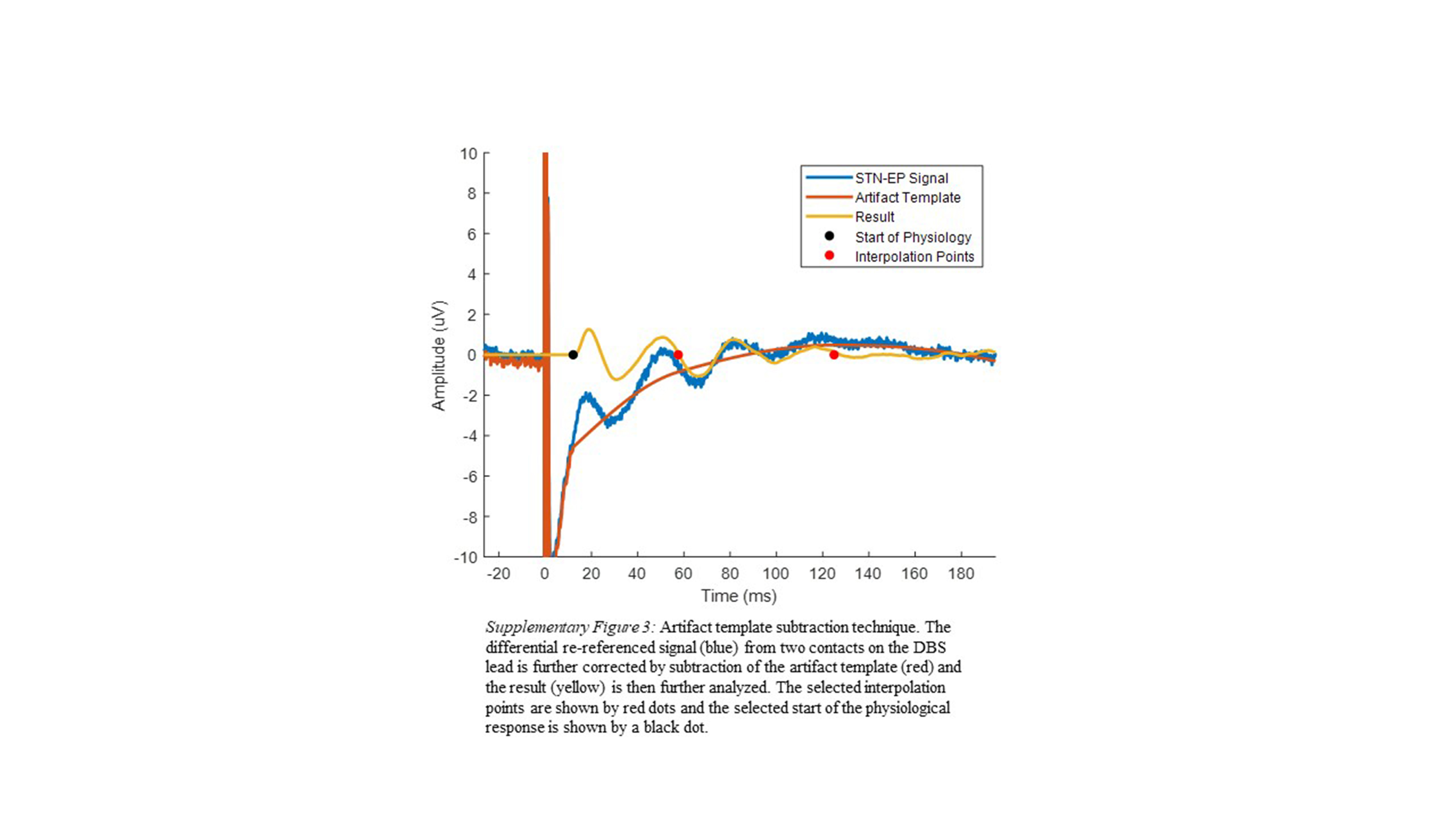

Supplement: Supplementary file 3 [file Image_3.JPEG]
